# A twelve-year longitudinal study of neuropsychological function in non-saturation professional divers

**DOI:** 10.1007/s00420-014-0991-0

**Published:** 2014-10-30

**Authors:** Rita Bast-Pettersen, Øivind Skare, Karl-Christian Nordby, Marit Skogstad

**Affiliations:** National Institute of Occupational Health, Oslo, Norway

**Keywords:** Diving, Prospective study, Neurobehavioral function, Benton Visual Retention Test, Q 16

## Abstract

**Objectives:**

Our main aim was to study the long-term neuropsychological effects of non-saturation diving. Further, we aimed to investigate whether neuropsychological performance was predictive of subsequent diving accidents and diving status.

**Methods:**

In this prospective longitudinal study, we enrolled 50 male diving students (mean age 25.3 years) at a diving school and followed them up six and 12 years later (43 and 37 divers, respectively). At each wave of the study, divers completed a comprehensive neuropsychological test battery and answered questionnaires on cumulative number of dives, incidents of decompression illness (DCI) and professional diver status.

**Results:**

At the 12-year follow-up, the divers reported a median number of 455 (range 40–5,604) cumulative dives. Cumulative number of dives was not associated with any adverse neuropsychological effects. However, divers with an incident of DCI performed worse in a memory test (Benton Visual Retention Test) and reported slightly more neuropsychiatric symptoms (Q 16). Diver students who performed well on a blindfolded memory test (tactual performance test) had an increased likelihood of becoming a professional diver 12 years later.

**Conclusions:**

The main findings in the present study support the view that asymptomatic non-saturation divers who have dived under controlled conditions do not have an increased risk of impaired nervous system function, at least not to an extent that can be detected with neuropsychological tests while they still are relatively young. The observed associations between a history of DCI and impaired results in a memory test and reporting of neuropsychiatric symptoms may be due to a nervous system effect caused by DCI. The diver students’ ability of problem-solving while they were blindfolded was predictive of their likelihood of becoming a professional diver 12 years later.

## Introduction

While the nervous system effects of saturation diving have been widely studied, there has been less focus on the effects on the nervous system related to non-saturation diving or diving in shallow water with air as the breathing gas. Nervous system effects can be caused by gas bubbles due to decompression illness (DCI).

Decompression illness (DCI) is an umbrella term for both arterial gas embolism (AGE) and decompression sickness (DCS). In AGE, alveolar gas or venous gas emboli (via cardiac shunts or via pulmonary vessels) are introduced into the arterial circulation (Vann et al. 2010). Decompression sickness (DCS) is caused by in situ bubble formation from dissolved inert gas (Vann et al. 2010). It typically afflicts divers during a poorly managed ascent from depth.

DCS has been characterized as type 1 (with musculoskeletal, skin, or lymphatic symptoms) or as type 2 (with more severe symptoms: neurologic, cardiorespiratory, audiovestibular), but it is now recognized that this differentiation is artificial (Grønning and Aarli 2011).

The risk of DCS increases with the presence and greater size of a *patent foramen ovale* (leakage from the right to the left part of the heart; Grønning and Aarli 2011). The clinical relevance of a patent foramen ovale is, however, unclear among asymptomatic young divers (Gempp et al. 2010).

Different diagnostic tools have been applied in order to study the nervous system effects of diving. Abnormal *EEG* has been reported in saturation divers (Todnem et al. 1991), but other studies among divers with histories of DCI, have not been able to demonstrate this result (Murrison et al. 1995). Reports from studies using magnetic resonance imaging (MRI) do not provide a clear picture. A study among professional experienced Navy bounce divers without DCI who were compared with non-diving colleagues, did not disclose correlations between neuropsychological test results, diving experience and number and size of MRI lesions (Cordes et al. 2000). A clinical study of 21 divers treated after acute decompression sickness in the CNS did not reveal cerebral lesions by MRI in any of the patients studied (Grønning et al. 2005).

### Neuropsychological studies of divers

Neurobehavioral and neuropsychological tests have been employed in several studies of nervous system effects in divers. These methods have been utilized in studies of divers with DCI/DCS (Peters et al. 1977; Curley et al. 1988), in studies of saturation divers (Værnes et al. 1987, 1988, 1989; Curley 1988), in studies of both saturation divers and non-saturation divers (Taylor et al. 2006), in studies of divers mainly using compressed air and in breath holding divers (Williamson et al. 1987; Tetzlaff et al. 1999; Bast-Pettersen 1999; Cordes et al. 2000; Leplow et al. 2001; Slosman et al. 2004; Ridgway and McFarland 2006; Gempp et al. 2010; Hemelryck et al. 2013), and in studies of heliox non-saturation (bounce) divers (Hodgson and Golding 1991).

Whether diving per se causes long-term health effects has also been discussed (Grønning and Aarli 2011; Wilmshurst 1997), but there is no clear consensus whether diving that does not result in DCI/DCS can cause nervous system effects (Tetzlaff et al. 1999; Bast-Pettersen 1999; Cordes et al. 2000).

Neuropsychological studies published before the present study was initiated, showed no consistent effects related to diving. Peters et al. (1977) in a study of divers with DCI reported deficient memory and reduced psychomotor and motor tempo. Curley et al. (1988) reported a variety of neuropsychological effects in a study with five cases. Værnes et al. (1987, 1988, 1989) in studies of saturation divers reported different neuropsychological outcomes, mainly in manual tests for tremor or motor speed. Curley (1988) reported no permanent effects in a study of Navy divers tested with a relatively broad test battery. In a study of current working and retired abalone divers, Williamson et al. (1987) reported poorer results in several tests for learning and memory, and they had poorer hand steadiness. On the other hand, they performed faster in a reaction time test. Hodgson and Golding (1991) applied a short computerized test battery covering reaction time, reasoning and memory. No significant decrement in performance was observed related to series of heliox non-saturation dives.

The available cross-sectional studies have been inconclusive. As far back as 1977, Peters and coworkers recommended obtaining baseline neurobehavioral results prior to employment. These guidelines have been repeated by several authors, who have also recommended the use of a longitudinal approach to the neurobehavioral testing of divers (Curley et al. 1988; Tetzlaff et al. 1999; Grønning and Aarli 2011) to avoid the potential biases and weaknesses of a cross-sectional design. To date, only a few studies using this approach have been published.

A diver must be able to work in the dark, without much visual aid. The ability to solve problems while blindfolded might be a method for testing future suitability for the diving profession. As diving is a high-risk profession, the ability to react quickly when dangerous situations arise can be very important to avoid injury. One aspect of this ability can be measured with reaction time tests. To date, studies using such tests to predict future diving activities have not been published.

## Objectives

The aim of this study was, prospectively, to examine the possible neuropsychological effects of non-saturation diving (bounce diving) on professional divers who mostly used air as their breathing gas. The hypothesis was that number of dives as well as a history of DCI could lead to impaired neuropsychological function. Due to the variety of test results in the available studies, a broad neuropsychological test battery was chosen.

A further aim was to investigate whether certain tests, administered to diving students, could predict their future diving activities, either in terms of incidents of DCI or whether the diver would become a professional diver. Our hypothesis was that subjects with a good ability to solve problems without the guidance of vision and/or subjects who had a fast reaction time would have an increased probability of becoming a professional diver and a decreased probability of incidents of DCI.

## Subjects and methods

During the years 1992–1994, students attending a professional diving school were invited to participate in a study of the possible health effects of diving. Ninety-three students from seven successive courses were invited, and 87 agreed to participate in the lung function study (Skogstad and Skare 2008). In addition, studies of auditory (Skogstad et al. 2009) and neuropsychological function were conducted. The neuropsychological study did not include students from the first and the last two courses. A neuropsychological examination is very time-consuming, and because of capacity reasons (time available), the neuropsychological study was limited to 50 subjects; a number that was considered adequate to provide sufficient statistical power when following up the divers.

The study was restricted to males because very few women attended the diving school. Furthermore, the study was restricted to Norwegian subjects. Because the students had passed a medical examination before attending the diving school, no further exclusion criteria were used.

The first examination was undertaken on the premises of the diving school, whereas the two follow-up examinations were conducted at the National Institute of Occupational Health in Oslo. All subjects that could be traced were invited to participate in each of the follow-up examinations. At the time of the first follow-up, 43 divers accepted to participate, giving a participation rate of 86 % after a follow-up time of 6 years. One diver, who did not participate in the 6-year follow-up, was included in the 12-year follow-up. At the time of the final follow-up, two divers had died (one in a diving accident), one had moved abroad, and one could not be traced. Among the 46 divers who were contacted before the last examination, 37 agreed to participate, giving a participation rate of 74 % of the original 50 subjects. The same licensed clinical neuropsychologist (the first author, RB-P) tested all the divers in the two follow-up sessions, and with a few exceptions, this psychologist also tested all the divers in the first examination. Among the 50 students who attended the first examination, 32 had served as a reference group in a study of long-term exposed construction divers (Bast-Pettersen 1999).

All subjects volunteered to participate in the study, and their written informed consents were obtained at all three examinations. The study was approved by the Regional Committee for Medical and Health Research Ethics, REK South–East, Norway.

### Diving exposure and background data

Prior to each of the three test sessions, the participants underwent a structured interview focusing on education, occupational history, smoking habits, alcohol consumption, accidents and illnesses. The structured interviews were non-blinded, as the psychologist who made the interviews also conducted the testing. All divers reported their diving activity in a questionnaire including number of dives (professional and recreational dives), before the first examination and between the successive examinations. Whether they worked as a professional diver or not (part time or full time) and whether they had experienced an episode of DCI were also recorded. The reporting of professional activity was based on the divers’ professional logbooks. Recreational diving was not properly registered in logbooks. Thus, the divers had to memorize to give their best estimate of number of dives. Most of their diving activity was performed with air as the breathing gas, either surface supported, or with scuba equipment. Four divers had become educated saturation divers during the follow-up period, but only one of them had performed deep-sea diving using other gas mixtures than air during the last 6-year follow-up period. Most of the dives were performed to shallow depths of ten meters or less (Skogstad and Skare 2008).

The subjects’ education was recalculated as the total number of years of formal schooling at each test session. Self-reported tobacco consumption was characterized by the use of smoked tobacco or smokeless tobacco in the form of moist snuff, called snus (Hugoson et al. 2012). The current alcohol consumption was calculated (L pure alcohol/year) based on the participants’ self-reported consumption of alcohol beverages (Hauge and Irgens-Jensen 1987).

### Test methods

To make the test results comparable over 12 years, the same versions of each of the tests were used in all three sessions, disregarding whether new versions of the same tests had been developed. This was the case with the Wechsler Adult Intelligence Scale (WAIS) tests, where the older Norwegian version (Engvik et al. 1978) was applied throughout the study. The test versions were identical to those used in a study of construction divers (Bast-Pettersen 1999). Each test session lasted between two and three hours.

*Neuropsychiatric symptoms*. The subjects were asked to complete a self-administered neuropsychiatric questionnaire (Q 16) (Lundberg et al. 1997; Bast-Pettersen 2006). The Q 16 was chosen as it has been applied among Norwegian occupational physicians and researchers in the field of occupational medicine in Norway since the early 1980s (Bast-Pettersen 1999, 2006, 2009; Bast-Pettersen et al. 2004). The Q16 requires the subject to answer “Yes” or “No” to 16 questions. The score was the total number (0–16) of symptoms.

### General intellectual ability

*Information* (WAIS) (Engvik et al. 1978). The subject is asked to answer a number of questions requiring general knowledge. The score was the number of correct responses.

*Block Design* (WAIS). The subject uses patterned blocks to copy different designs shown on a picture. The score was based on the number of correct solutions within the time limits, with a bonus added for fast responses.

### Tests for visuomotor processing speed

*Digit Symbol* (WAIS). This test requires the subject to recode symbols to digits in the course of 90 s. The score was the number of correct symbols recoded.

*The Trail Making Test* (Reitan and Wolfson 1985; Strauss et al. 2006). The subject is required to connect, by making pencil lines, 25 encircled numbers randomly arranged on a page in their proper order (part A) and 25 encircled numbers and letters in alternating order (Part B). The scores were the amount of time used to complete each section of the test (in s).

### Attention span and working/visual memory

*Digit Span* (WAIS). The subject is required to repeat as many digits as possible after an oral presentation, either in the same order as they were presented (Digits Forwards) or in the reverse order (Digits Backwards). The scores were maximum number of digits repeated.

*Benton Visual Retention Test* form C, administration A (Sivan 1992). This test employs 10 cards that have increasingly complex visual designs. The subject is required, immediately after a 10 s viewing of a card, to reproduce its design from memory on blank paper. The number of correct reproductions and the number of errors were recorded. The evaluation of the Benton test drawings was performed by a student who was trained to use the scoring criteria given in the test manual (Sivan 1992).

### Motor speed/manual dexterity

*Grooved Pegboard Test* (Lafayette Instrument Company). This test consists of a small board containing a 5 × 5 set of slotted holes angled in different directions and 25 pegs with a ridge along one side. The subject is required to rotate the peg into position for correct insertion as quickly as possible. This is a test for manual dexterity and motor speed. The score was time to completion in s for each hand.

### Tests for reaction time

The NES2 version 4.51 (Letz et al. 1996) was used for all three sessions. The tests were run in MS-DOS with a computer that was kept through the years for this project.

*Simple Reaction Time test* (SRT) (NES2 version 4.51) (Letz et al. 1996) requires the subject to press a button as quickly as possible when a square appears on a computer screen. Ninety reaction times were recorded. The first 10 trials were regarded as practice and were discarded. The score was the individual’s mean reaction time (RT) in milliseconds (ms).

*Continuous Performance Test* (CPT) (NES2 version 4.51) (Letz et al. 1996). Several different letters flash briefly on the screen for approximately 50 ms at a rate of one per second for five minutes. The subject is required to press a button upon seeing an “S” flash on the screen, but not for any other letter. The number of RTs collected was 60, and the first twelve were discarded. The score was the response latency (mean RT) in ms.

### Spatial skills

*Tactual Performance Test* (TPT) (Psychological Assessment Resources, PAR). While blindfolded, the subject is presented with blocks of differing shapes and a form board with matching shaped holes. The subject is required to insert the blocks into the board as quickly as possible and then to draw the board’s organization from memory, thus testing the memory for the form (TPT-Memory) and localization (TPT-Localization) of the blocks (Strauss et al. 2006). The scores were the time to complete the test and the number of shapes remembered. Due to restricted testing time in the follow-up study, this time-consuming test was only applied in the baseline study, and the results were used in a model to test for prediction of future diving activity.

### Statistics

Using a linear mixed model (Fitzmaurice et al. 2011), separate analyses were made for each test outcome and each diving parameter, giving a total of 28 analyses (Table 3). In the analyses, adjustments were made for age, self-reported alcohol consumption and the use of tobacco (smoking or snus). Adjustments were also made for the level of education, measured as years of schooling, except for the tests CPT and Trail Making Test B, where reading difficulties were of major relevance and was instead chosen as a covariate. Reading difficulties was a baseline covariate; all other covariates were time varying variables. The Q16 was only adjusted for age.

In order to control for the possible improvements in test performance due to repeated exposure to the tests, the test sequence number (1, 2 or 3 for first, second or third test session), considered as a categorical variable, was also adjusted for in the analyses.

To enhance power, adjustment was only obligatory for age, test sequence number, reading difficulties/years of education. Alcohol consumption and nicotine consumption (cigarettes or snus) could be excluded from the model. For each analysis, this was done by means of a stepwise backwards selection procedure, using the Akaike information criterion.

The data contained some missing values for the covariates included in the model, i.e., six missing values for the use of nicotine at the baseline and three missing values for the alcohol consumption variable. To include all the data in our analysis, the linear mixed model was combined with multiple imputation. The imputation was based on the longitudinal correlation of smoking status and alcohol consumption, the dependency of these covariates on the baseline values of age and test outcome, and the number of dives if it was included in the analysis.

A mixed model includes both fixed and random effects. Fixed effects model the systematic effects of covariates such as age and educational level, while random effects model the dependency structure of data. The final model takes into account the dependency of the repeated observations by adding random intercepts for each subject. The mixed model can be represented formally as *y*_*ij*_ = *x*_*ij*_^*T*^ *β* + *u*_*i*_ + *ɛ*_*ij*_, where *y*_*ij*_ is the observed test outcome of diver i and replication j, and $x_{ij}^{T}$ is a vector of regressors linking the observations to the fixed effects, *β*. Furthermore, *u*_*i*_ represents independent and identically normally distributed random effects with a mean 0 and variance *σ*_*u*_^2^, while *ɛ*_*ij*_ are independent and identically distributed normal random variables with a mean 0 and variance *σ*_*ɛ*_^2^. Variance structures of varying complexity were compared using Wald tests. For the test WAIS Block Design, the residual variance was allowed to differ between years, while for CPT the between-subjects variance was allowed to differ between years. Different transformations of the outcome variables were compared, and a log-transformation (log_10_) gave the best fit to the normal distribution, as seen by a quantile–quantile plot, for the following outcome variables: Q16, Pegboard non-dominant hand, Trail Making Test B, SRT, and CPT. The other outcome variables were not transformed.

Fisher’s exact mid *p* test was used to analyse the questions on Q 16 (Table 4).

A paired *t* test was used to compare the test results of the divers between the first and the last test session (Table 5).

For the analyses of associations between the baseline results of the TPT and future diving parameters as well as for the reaction time measures and future diving parameters (Table 6), separate logistic regression analyses were made for each combination of test variable (exposure) and diving parameter (outcome).

The analyses were first made without adjusting for the covariates. Adjustments were then made for age and years of education when using the predictors TPT-Total Time, TPT-Memory, TPT-Localization, and SRT. For the analyses using the predictor CPT, adjustments were made for age and reading difficulties.

Linear mixed models with multiple imputation were analyzed in R (www.r-project.org) using the packages nlme and mix. The linear regressions were also analyzed in R; all other analyses were performed with SPSS v 15.0 for Windows (SPSS, IL, USA). The level of significance was set at *p* = 0.05.

## Results

Table 1 shows the background data and test results for the 50 students who were included in the first (baseline) study.

**Table 1:** Background data and neuropsychological test results for the 50 diving school students included in the baseline study

	Mean	SD	Min–max
Age	25.3	4.3	18–35
Years of education	12.1	1.5	9–16.5
Self-reported alcohol consumption (L/year)	4.96	3.6	0–14.7
Prevalence of smokers (%)	51.4	0.5	
Prevalence of smokers or use of smokeless tobacco (snus) (%)	59.5	0.5	
Cigarettes/week	66	65	0–210
Prevalence right-handers	86		
Prevalence of reading difficulties	20		
Self-reported neuropsychiatric *symptoms* (Q16)^b^	1.2	1.5	0–7
*Tests for intellectual ability*			
WAIS Information number correct (raw score)^a^	20.7	3.9	11–28
WAIS Block Design (raw score)^a^	42.2	5.8	28–48
*Tests for visuomotor processing speed*			
WAIS Digit Symbol (number correct)^a^	58.2	9.7	40–78
Trail Making Test A (s)^b^	23.5	7.1	12–45
Trail Making Test B (s)^b^	68.6	35.3	29–236
*Attention span/working/visual memory*			
WAIS Digit Span forwards^a^	6.6	1.2	4–9
WAIS Digit Span backwards^a^	5.6	1.5	3–8
Benton Visual Retention Test correct^a^	8.4	1.0	6–10
Benton Visual Retention Test errors^b^	2.0	1.4	0–6
*Motor speed/manual dexterity*			
Pegboard dominant hand (s)^b^	59.4	6.5	47–75
Pegboard non-dominant hand (s)^b^	65.3	10.9	49–114
*Spatial skills/spatial memory*			
Tactual Performance Test (TPT) total time (min)^b^	10.7	4.0	3.2–26.1
Tactual Performance Test (TPT) Memory (number correct)^a^	8.8	0.9	7–10
Tactual Performance Test (TPT) Localization (number correct)^a^	7.7	1.5	4–10
*Tests for Reaction Time (NES2)*			
Simple Reaction Time (SRT) (ms)^b^	222	19	184–276
Continuous Performance Test (CPT) (ms)^b^	373	30	323–444

^a^A higher value is a better score

^b^A lower value is a better score

Table 2 shows the diving parameters, number of dives and prevalence of DCI for the 50 divers in the baseline study, the 43 divers in the 6-year follow-up and the 37 divers in the final follow-up. While the divers had performed a median number of 58 (0–1,500) dives before entering the diving school, 15 had never dived. Most of the diving activity was performed during the first 6-year period. Eleven divers reported a history of DCI at the 6-year follow-up (including one who had an incident of DCI before attending the diving school). Ten of the divers with a history of DCI attended the final follow-up. One diver died from a diving-related accident after the second follow-up. He reported no diving-related accidents before the fatal one.

**Table 2:** Diving exposure parameters at baseline, 6- and 12- year follow-up

	Mean (SD)	Median	Min–max
Baseline (*N* = 50)			
Number dives before diving school	216 (326)	58	0–1,500
Prevalence DCI before diving school	0.02		
Six years (*N* = 43)			
Number dives	514 (654)	298	40–3,654
Prevalence DCI	0.26		
Twelve years (*N* = 37)			
Number dives	767 (1,078)	455	40–5,604
Prevalence DCI	0.27		

Table 3 shows the neuropsychological test results related to the number of dives and to reporting an incident of DCI.

**Table 3:** Neuropsychological test results related to number of dives and lifetime history of DCI. *N* = 50, 43 and 37 for the first, second and third part of the longitudinal follow-up^a^

Test outcome	Exposure	Estimate	Lower	Upper	*p* value
Self-reported *symptoms*, Q16^c, d^	Number dives^d^	−0.02	−0.08	0.05	0.61
	Incident of DCI	0.16	0.02	0.29	0.02
*Tests for intellectual ability*					
WAIS Information^b^	Number dives^d^	−0.54	−0.98	−0.10	0.02
	Incident of DCI	0.26	−0.71	1.23	0.60
WAIS Block Design^b^	Number dives^d^	−0.66	−1.65	0.33	0.19
	Incident of DCI	0.26	−1.48	2.01	0.77
*Tests for visuomotor processing speed*					
WAIS Digit Symbol^b^	Number dives^d^	0.49	−0.86	1.85	0.47
	Incident of DCI	1.57	−1.31	4.45	0.29
Trail Making Test A^c^	Number dives^d^	−0.49	−2.19	1.21	0.57
	Incident of DCI	−0.70	−4.30	2.90	0.70
Trail Making Test B^c, d^	Number dives^d^	0.00	−0.03	0.04	0.84
	Incident of DCI	−0.07	−0.14	0.01	0.07
*Attention span/working/visual memory*					
WAIS Digit Span—forward^b^	Number dives^d^	0.00	−0.23	0.23	0.99
	Incident of DCI	−0.01	−0.50	0.49	0.98
WAIS Digit Span—backward^b^	Number dives^d^	0.03	−0.25	0.31	0.84
	Incident of DCI	−0.44	−1.03	0.14	0.13
Benton Visual Retention Test Correct^b^	Number dives^d^	−0.07	−0.33	0.19	0.59
	Incident of DCI	−0.71	−1.23	−0.18	0.008
Benton Visual Retention Test Errors^c^	Number dives^d^	0.20	−0.16	0.55	0.28
	Incident of DCI	1.29	0.58	2.00	0.0003
*Motor speed/manual dexterity*					
Pegboard dom hand^c^	Number dives^d^	−1.40	−2.83	0.03	0.06
	Incident of DCI	−2.27	−5.32	0.79	0.15
Pegboard non-dom hand^c d^	Number dives^d^	−0.01	−0.02	0.01	0.40
	Incident of DCI	−0.01	−0.04	0.01	0.35
*Tests for reaction time (NES2)*					
Simple Reaction Time (SRT)^c d^	Number dives^d^	0.00	−0.01	0.00	0.60
	Incident of DCI	−0.01	−0.03	0.00	0.04
Continuous Performance Test (CPT)^c d^	Number dives^d^	−0.01	−0.01	0.00	0.16
	Incident of DCI	−0.01	−0.03	0.01	0.20

^a^Linear mixed model. For the Q 16, adjustments were only made for age. All other variables were in addition to age-adjustment, either adjusted for years of education or for reading difficulties (Trail Making Test A and B and NES CPT). In addition, the Digit Span Forwards, the Pegboard test and the Trail Making Test A were corrected for use of nicotine consumption

^b^A higher value is a better score

^c^A lower value is a better score

^d^Log transformed (log_10_)

An increase in the number of dives was associated with a lower score on the WAIS Information test.

The divers reporting an incident of DCI reported more symptoms on the Q 16 (*p* = 0.023), and they had significantly weaker test results in the memory test, Benton VRT, both with respect to number correct (*p* = 0.008) and with respect to number errors (*p* = 0.0003). On the other hand, they performed significantly better in the reaction time test, SRT (*p* = 0.04).

Sixteen divers reported being a professional diver (many only part-time) at the 12-year follow-up. None of the test results at the 12-year follow-up were related to whether the participants reported a professional status as diver (not tabulated).

Self-reported symptoms assessed with the Q16 are shown in Table 4. The divers with incident of DCI did not answer “yes” to the questions related to memory complaints (6, 7, 11, 13, 14) more often than divers without DCI. The question with largest contrast between the divers with and without DCI was “do you have headache once a week?” (*p* = 0.12).

**Table 4:** Self-reported neuropsychiatric symptom, the Q16. Results for the divers included in the 12-year follow-up

	Question	All divers (*N* = 37)	Divers without DCI incident (*N* = 27)	Divers with DCI incident (*N* = 10)	Diff. in positive answers (%) divers with and without DCI incident
		Positive answers (%)	Positive answers (%)	Positive answers (%)	*p* value^a^
1	Are you abnormally tired?	5.4	7.4	0	0.53
2	Do you have palpitations even when you are not exerting yourself?	8.1	3.7	20	0.19
3	Do you often have a painful tingling in some part of your body?	2.7	0	10	0.27
4	Do you often feel irritated for no particular reason?	10.8	7.4	20	0.34
5	Do you often feel depressed for no particular reason?	8.1	3.7	20	0.19
6	Do you often find it difficult to concentrate?	27	29.6	20	0.60
7	Do you have a short memory?	37.8	40.7	30	0.58
8	Do you perspire for no particular reason?	2.7	3.7	0	0.73
9	Do you have any problems with buttoning and unbuttoning?	0	0	0	NA
10	Do you generally find it hard to grasp the meaning when reading newspapers and books?	8.1	7.4	10	0.8
11	Have your relatives told you that you have a short memory?	29.7	29.6	30	0.97
12	Do you sometimes feel a “weight” on your chest?	5.4	3.7	10	0.54
13	Do you have to make notes about what you must remember more often than you think is normal?	13.5	14.8	10	0.77
14	Do you often have to go back and check things you have done, such as turned off the stove, locked the door, etc.?	27	25.9	30	0.80
15	Do you have a headache at least once a week?	13.5	7.4	30	0.12
16	Are you less interested in sex than you think is normal?	0	0	0	NA

Percentage answering “yes” to each question, for all of 37 divers, for the divers without and with DCI incident. Lower and upper CI for difference in percentage of positive answers between divers with and without DCI incident

^a^Fisher’s exact mid *p* test

Table 5 shows the unadjusted test results at baseline and at the six- and 12-year examinations for the 37 participants who attended the last follow-up. For the 37 subjects who attended the last test session, the number of self-reported symptoms (Q 16) increased statistically significant during the 12-year period (*p* = 0.015). While the self-reported alcohol consumption increased from 5 to 7 L/year (*p* = 0.08), the prevalence of smokers decreased considerably, from 51 to 32 % (*p* = 0.03). However, when taking into consideration the use of nicotine from cigarettes and smokeless tobacco (snus) together, the decrease in nicotine consumption over the 12 years was no longer significant.

**Table 5:** Neuropsychological test results for the 37 participants at the baseline, 6- and 12- year follow-ups, unadjusted results

	Baseline *N* = 37	6 year follow-up *N* = 36	12 year follow-up *N* = 37	Mean diff. baseline versus 12-year follow-up	*P* ^c^
	Mean (SD)	Mean (SD)	Mean (SD)		
Age	25.7 (4.3)	31.5 (4.1)	36.9 (4.4)	+11.2	
Years of education	12.2 (1.5)	13.3 (1.6)	14.1 (1.9)	+1.9	
Cumulated number of dives during lifetime (median; min–max)	60 (0–1,000)	302 (40–3,654)	455 (40–5,604)	+397	
Years diving	3.6 (4.5)	8.4 (5.1)	12.2 (6.7)	+8.6	
Self-reported alcohol consumption (L/year)	5.01 (3.2)	4.93 (4.7)	7.03 (7.6)	+2.02	0.08
Smokers (%)	51.4	52.8	32.4	−18.9	0.03
Smokers/users of smokeless tobacco (%)	59.5	61.1	51.4	−8.1	0.32
Cigarettes/week	62 (61)	56 (64)	32 (59)	−30	0.005
Prevalence reporting incident of/DCI (%)	2.7	24.3	27		
Self-reported *symptoms*, (Q16)^b^	1.2 (1.5)	1.3 (1.8)	2.0 (2.0)	+0.8	0.015
*Test for intellectual ability*					
WAIS Information, number correct^a^	21.4 (3.8)	21.9 (3.6)	22.4 (3.4)	+1.0	0.001
WAIS Block Design^a^	43.5 (4.9)	43.8 (4.8)	44.8 (3.3)	+1.3	0.012
*Tests for visuomotor processing speed*					
WAIS Digit Symbol^a^	59.3 (9.3)	61.0 (8.5)	60.2 (9.6)	+1. 0	0.22
Trail Making Test A^b^	23.1 (7.4)	22.3 (8.0)	22.9 (7.4)	−0.2	0.90
Trails Making Test B^b^	61.4 (24.1)	61.4 (23.5)	64.0 (31.9)	+2.6	0.56
*Attention span/working/visual memory*					
WAIS Digit Span—forward^a^	6.8 (1.1)	6.7 (0.9)	7.1 (1.1)	+0.3	0.86
WAIS Digit Span—backward^a^	5.8 (1.4)	5.7 (1.4)	5.9 (1.2)	+0.1	0.51
Benton Visual Retention Test Correct^a^	8.4 (0.9)	8.3 (1.1)	8.6 (1.1)	+0.2	0.26
Benton Visual Retention Test Errors^b^	1.9 (1.2)	2.1 (1.5)	1.6 (1.5)	−0.3	0.27
*Motor speed/manual dexterity*					
Pegboard, dominant hand^b^	59.6 (6.8)	57.6 (6.9)	57.9 (10.2)	−1.6	0.33
Pegboard, non-dominant hand^b^	65.6 (11.3)	62.0 (8.6)	64.8 (9.4)	−0.8	0.66
*Spatial skills/spatial memory*					
Tactual Performance Test total time^b^	10.4 (4.5)	–	–	–	–
Tactual Performance Test Memory^a^	8.9 (0.9)	–	–	–	–
Tactual Performance Test Localization^a^	8.0 (1.4)	–	–	–	–
*Tests for reaction time (NES2)*					
Simple Reaction Time (SRT)^b^	222 (20)	221 (19)	222 (17)	0	0.91
Continuous Performance Test (CPT)^b^	372 (28)	364 (28)	367 (30)	−5	0.34

^a^A higher value is a better score

^b^A lower value is a better score

^c^Paired *t* test for difference between baseline data versus 12 years follow-up

Most of the neuropsychological test results (tests for attention span/working memory, processing speed and manual dexterity) were remarkably stable over the 12-year period. However, during this 12-year period, the participants improved their performance on the WAIS tests, Information (*p* = 0.001) and Block Design (*p* = 0.012).

Table 6 shows the estimates of the TPT measures and reaction time measures at baseline, related to whether the students later became professional divers and whether they later experienced an incident of DCI.

**Table 6:** Prediction of future diving parameters based on baseline measures for the Tactual Performance Test (TPT) and reaction times (SRT and CPT) ^a^

Outcome	Predictor	Unadjusted				Adjusted			
		Estimate	Lower	Upper	*p* value	Estimate	Lower	Upper	*p* value
Professional diver at T12	Tactual Performance Test (TPT)-total Time^b^	−0.12	−0.33	0.04	0.20	−0.13	−0.37	0.06	0.23
	Tactual Performance Test (TPT)-Memory^c^	1.20	0.31	2.30	0.016	1.30	0.34	2.40	0.016
	Tactual Performance Test (TPT)-Localization^c^	0.31	−0.14	0.84	0.20	0.42	−0.08	1.00	0.13
	Simple Reaction Time (SRT)^b,d^	0.011	−0.023	0.047	0.53	−0.0039	−0.047	0.037	0.85
	Continuous Performance Test (CPT)^b,d^	−0.0042	−0.031	0.021	0.74	−0.0043	−0.033	0.023	0.76
Incident of DCI	Tactual Performance Test (TPT)-total time^b^	−0.07	−0.28	0.1	0.47	−0.03	−0.25	0.15	0.73
	Tactual Performance Test (TPT)-Memory^c^	−0.43	−1.30	0.36	0.29	−0.59	−1.50	0.26	0.19
	Tactual Performance Test (TPT)-Localization^c^	−0.23	−0.71	0.24	0.34	−0.35	−0.91	0.16	0.19
	Simple Reaction Time (SRT)^b,d^	0.0073	−0.031	0.047	0.71	0.0029	−0.042	0.047	0.89
	Continuous Performance Test (CPT)^b,d^	−0.0017	−0.032	0.026	0.90	−0.0021	−0.032	0.026	0.89

^a^Logistic regression analysis, unadjusted estimates (left) and estimates adjusted for age and years of education at baseline (for CPT reading difficulties)

^b^A lower value is a better score

^c^A higher value is a better score

^d^Log transformed (log_10_)

The ability to remember the presented TPT figures while blindfolded was predictive of the likelihood of becoming a professional diver 12 years later (*p* = 0.016). The reaction time measures at the first examination did not predict future diving activity. None of the test results at baseline did predict future incidents of DCI. For the outcome of DCI, an analysis with additional adjustments for number of dives and accidents per number of performed dives was performed, but the associations with the TPT measures and the reaction time tests remained nonsignificant (not tabulated).

## Discussion

This longitudinal study investigated the associations between non-saturation diving and neuropsychological function. The main findings were that the number of performed dives was not associated with impaired test results and that divers with a history of DCI performed less well in a memory test and they reported more neuropsychiatric symptoms. The results of a test that the students solved while blindfolded were predictive of their likelihood of becoming a professional diver 12 years later.

The participation rate in the follow-up study was high: 86 % after 6 years and 74 % of the original group after 12 years (77 % of those still alive), thus reducing the impact of selection bias. Although several researchers have agreed with the recommendations given by Peters and coworkers (1977), to obtain baseline neurobehavioral results prior to employment, this is, to our knowledge, the first available neuropsychological study that has followed divers for several years (12 years), starting when the participants still were in school.

### Effects related to numbers of dives

In the final follow-up, the divers had performed a median of 455 dives, ranging from 40 to 5,604 dives. This was considerably fewer dives than in the Norwegian study of construction divers, where the average number of dives was reported to be 4,087, ranging from 450 to 13,200 dives (Bast-Pettersen 1999).

With one exception, no impairment in test results was associated with the number of dives. The performance in the WAIS Information test was reduced related to the number of dives, but not with a history of DCI. The weaker performance on WAIS Information among the divers who had performed more dives was most likely not an effect of diving. The WAIS Information test is often used as a marker of “premorbid function.” The association with number of dives was more likely due to the choice to exclude diving as a profession among subjects with a more theoretical orientation, which the WAIS Information test is supposed to measure.

The finding that no nervous system effects were associated with the number of dives (diving *per se*) is in accordance with several other studies. Cordes et al. (2000) reported that there were no significant correlations between neuropsychological test results and diving indices in a group of military compressed-air divers without DCI, if the diving was conducted under controlled conditions. Ridgway and McFarland (2006) found normal neuropsychological test results in a group of breath-hold divers. Other studies have found associations between diving and neuropsychological effects. In a study that covered both saturation divers and non-saturation divers, mixed bounce diving and surface oxygen decompression diving were associated with poorer memory performance. DCI did not explain any of these associations (Taylor et al. 2006). A large study of UK divers not undergoing litigation, demonstrated that divers were more prone to report symptoms of any kind, but especially in the form of “forgetfulness or loss of concentration” regardless of their state of health (Ross et al. 2007).

### Effects related to DCI

Altogether, eleven divers reported an episode of DCI; ten of them were examined in the 12-year follow-up. An increased number of self-reported neuropsychiatric symptoms was associated with a history of DCI but not with the number of performed dives. The divers with incident of DCI did not report memory problems on the Q 16. The finding of impaired results in a memory test without reporting memory problems is in accordance with a study of self-reported conceptions of memory and concentration, which reported that self-reported cognitive abilities can be trusted to only a limited degree (Bast-Pettersen 2006). Increased number of symptoms was reported by Trevett et al. (2010). Irgens et al. (2007) reported a reduced health-related quality of life among former North Sea divers with a history of DCS compared with divers without DCS. However, a large proportion of these divers with a history of DCS were involved in a litigation process, which might influence their rating of their quality of life.

In the present study, a history of DCI was associated with a reduced performance in the memory test, Benton Visual Retention Test, with an increase of 1.3 (95 % CI 0.6, 2.0) errors and a decrease of 0.7 (95 % CI −1.2, −0.2) correct scores (Table 3). Reporting a history of DCI was associated with increased performance on SRT, with a difference in reaction time of −7.3 (95 % CI −13.9, −0.5) ms.

The Benton Visual Retention Test is a widely used memory test. The version used in this project allows a 10 s exposure to each card with immediate recall by drawing. The test covers several cognitive aspects but a common view is that it primarily is a test of memory skills and secondarily a test of attention span and perceptual-analytical ability. Executive control processes appear also to play a role in test performance (Strauss et al. 2006). The observed increase of 1.3 number of errors associated with DCI, corresponds closely to the average increase in number of errors between the age group 30–39 and the age group 50–59, since, according to the norms, the expected increase in number of errors is 1.34 errors between those age groups (Sivan 1992). The observed decrease in number of correct designs of 0.7 associated with DCI was in the same way close to the expected difference in average performance between the age group 30–39 and the group 50–59, where the average decrease according to the norms is 0.8 correct designs. In an oversimplified way, it could be said that having a history of DCI resulted in a reduced performance in a memory test corresponding to almost 20 years of aging.

However, we cannot completely rule out the possibility that the association between impaired test results and a history of DCI could be due to an unmeasured underlying predictor. The first author has, as a clinician, observed that in contrast to the Digit Span test where the psychologist controls the patient’s attention by presenting a new digit every second, response style can be quite important when performing the Benton Visual Retention Test. That is, if the person has a tendency to be a little impatient and do not utilize the10 s time period to check all the details, but ignores the complexity of the task, then his response style can affect the results.

In many ways, the Benton and the SRT measure opposite cognitive qualities. There is no time pressure when answering the paper-and-pencil test Benton Visual Retention Test, and the subject can actually correct his drawing if he thinks he has made an error. The Simple Reaction Time test, on the other hand, assesses the response time in milliseconds. The SRT rewards the ability to react quickly without reflecting over whether you might have made mistakes. This tendency might be associated with risk-taking behavior, which in turn could lead to an increased chance of getting into an accident, and then an increased risk of having an incident of DCI. If this was the case in the present study, this response style could represent a hypothetical unmeasured independent predictor (Hernan and Robins 2013), for example a tendency to react a bit quick without checking all details.

The most important argument against this explanation is that we actually measured the reaction times at baseline and found that the reaction time measures at baseline did not predict future diving activity including incidents of DCI.

A more plausible explanation could be that the observed associations between test results and incidents of DCI could reflect a change in cognitive qualities due to DCI, resulting in a tendency to respond quick but in a more superficial way, without checking all details.

Several studies have reported memory problems related to DCI. Værnes et al. (1989) reported impaired results in a memory test in a group of saturation divers who had experienced an accident as a diver. Peters et al. (1977) reported impaired memory, tested with the Wechsler Memory Scale, in divers with DCI.

In contrast to our study, Værnes et al. (1989) also found weaker performance in the Trail Making Test B and in the Pegboard test among saturation divers with a previous diver-related accident. Peters et al. (1977) found impaired results in several neuropsychological tests, including reduced IQ.

The divers in the present study had a fast reaction time, with an average SRT of 222 ms in the baseline study as well as in the last follow-up, and reporting an incident of DCI was associated with a slightly increased performance. Hemelryck et al. (2013) found a faster reaction time in a group of SCUBA divers compared with referents. However, our finding of improved results in the SRT seems to be in contrast to the findings of Kowalski et al. (2011), who found prolonged reaction times in very experienced scuba divers (military personnel) without DCS. The finding is also in contrast to the findings in the Norwegian study of construction divers (Bast-Pettersen 1999), where a slightly prolonged reaction time (238 ms) was ascribed to extensive non-saturation construction diving. The construction divers in the 1999 study were older (mean age 39.8 years), and they had performed more dives (median number 1,750, compared to 455 in the present study). In a control group of male industrial workers, an average reaction time of 233 ms was found by using the same test equipment as in the present study (Bast-Pettersen et al. 2004).

All in all, the divers in this study had very good results in the Simple Reaction Time test. We have no explanation for the observed association between fast responsiveness and DCI. As previously mentioned, even if we consider it to be less likely, an unmeasured underlying predictor (Hernan and Robins 2013) cannot be ruled out completely.

### Test results and background data related to a 12-year time span and to repeated measurements

It is noteworthy that for many of the test parameters, there were almost identical results during the 12 years, indicating the stability over time of many neuropsychological tests (Table 5). Self-reported symptoms measured with the Q 16 increased during the 12-year follow-up. The actual number of symptoms (2.0) for the group in the last follow-up was comparable to what has been reported by reference groups in other studies of male manual workers (Bast-Pettersen et al. 2004; Bast-Pettersen 2009).

The increase of 2.0 L/year in self-reported *alcohol consumption* was in accordance with the average consumption and increase in Norwegian alcohol consumption over the same years, Norway being the Nordic country with the lowest self-reported alcohol consumption (SIRUS, http://www.sirus.no/eng). The prevalence of smokers decreased considerably, but when taking into consideration the use of nicotine either as smoked or smokeless tobacco, the reduction was smaller, from a prevalence of 59.6 to a prevalence of 51.4.

A phenomenon that has to be taken into consideration when a study utilizes repeated exposure to test materials is the practice effect, which refers to the impact of repeated exposure to a test, which may lead to improvements in test performance (Lezak et al. 2012; McCaffrey et al. 2000). In general, neuropsychological tests with a speeded component, those requiring an infrequently practiced response, or have a single, easily conceptualized solution, are most susceptible to the effects of practice (Lezak et al. 2012; McCaffrey et al. 2000). Practice effects tend to be most pronounced with repetition of the same test, but sheer test taking exposure can improve subsequent performances (McCaffrey et al. 2000). Although widely known, the literature offers little guidance on the interpretation and how to handle the practice effects. Improved test scores in tests for intellectual ability have been reported in many studies using intelligence tests (McCaffrey et al. 2000). In the mixed model analysis, the possible practice effect was taken into consideration by adjusting for the test sequence number (Table 5).

In the present study, the divers improved their results in the two subtests for intellectual ability (the WAIS subtests Information and Block Design). Few studies have retested a study group with 12 years between the first and the last session. The improved results in tests for intellectual ability might be due to a practice effect.

Studies on the neurobehavioral effects of diving seem to have given little attention to a possible practice effect. Curley (1988) applied neuropsychological tests in experiments with Navy saturation divers over a 3-year period and administered the tests within 1 week before and after the dives. During most of the experiments, the divers performed better in the second test session, which might be due to a practice effect (McCaffrey et al. 2000).

### Prediction of future diving activity based on test results at baseline

A diver must be able to work in the dark, without much visual aid. Therefore, one of the aims was to study whether the ability to solve problems while blindfolded could predict future diving parameters. The finding that the students who were able to remember most wooden shapes presented to them while blindfolded had an increased likelihood of becoming a professional diver 12 years later is of interest (Table 6). This might indicate a self-selection to the diving profession of divers with a good ability to solve problems without the guidance of vision. We are not aware of other studies trying to predict future diving activity with this kind of neuropsychological tests. Other researchers have studied spatial abilities among divers, but they have focused on spatial abilities as an effect of diving either with the TPT (Værnes et al. 1989) or by using a locomotor maze to study whether spatial orientation was impaired in construction divers (Leplow et al. 2001). The reaction time measures at baseline were not associated with future diving parameters.

### Aspects of validity

One of the strengths of this longitudinal study is the long follow-up time with a high participation rate. At the time of the first follow-up, 43 divers accepted to participate, giving a participation rate of 86 percent after a follow-up time of 6 years. In the last follow-up, there was a participation rate of 74 % of the original 50 subjects.

The high participation rates suggest that selection bias is not expected to have a large impact on results. Further, a mixed model approach is robust to baseline differences and to dropout if the mechanism of missing observations at follow-up is missing at random. The high participation rate is remarkable considering that participating in the study was demanding of the participants. The test sessions were quite long, and several participants had to travel a long distance to participate in the follow-up studies. Furthermore, men are less likely to participate in scientific studies than women (Galea and Tracey 2007).

The same certified clinical neuropsychologist (RB-P) tested all the divers in the two follow-up sessions and, with a few exceptions, all the divers in the first examination. The same test equipment was used across the test sessions, thus allowing stability in the way the test results were obtained.

In recent years, attention has been drawn to the concept of cognitive malingering defined as the intentional exaggeration or fabrication of illness or disability motivated by external incentives (Greve et al. 2006) and to the concept of suboptimal performance (van Hout et al. 2003). This phenomenon has been discussed as a methodological problem in studies of divers involved in a litigation process (Ross et al. 2007). However, patients in a litigation process are very different from the healthy young men in our study. None of the divers in the present study were involved in a litigation process. The divers were at the start of their diving careers, and they were anxious not to lose their certificate of medical fitness. Therefore, they were highly motivated to perform at their best, which was also illustrated clearly in the reaction time performances. Therefore, no validity tests were considered necessary in the present study.

The possibility that the impaired results in a memory test in divers with DCI could be due to somatoform disorders can also be ruled out. Patients with somatoform disorders often report chronic unexplained pain and fatigue. They also tend to be depressed, and many report problems with memory and concentration. And as shown in Table 4, the divers did not have memory or concentration complaints. In a psychological study of Gulf War veterans with and without Gulf War unexplained symptoms (Storzbach et al. 2000), reaction time tests were more affected than memory tests among the case subjects with unexplained symptoms.

The relatively small number of subjects participating in this study represents a weakness of the study. Due to the moderate sample size, only the most important covariates: age, education or reading difficulties and practice effect were obligatory adjusted for. Smoking habits and alcohol consumption were only included if they were retained by the backward selection procedure described in the statistics section. The number of observations was 130, but, due to the dependency in data, the effective number of observations was somewhat less. The sample size was considered sufficient for managing the variables added to the model.

The limited number leads to even smaller subgroups; only 27 % of the divers reported a history of DCI. Another weakness of the study is that DCI is not well defined, and that stratification on type 1 and type 2 DCS has not been undertaken. However, neurologic symptoms may be overlooked (Vann et al. 2010) once for instance when pain in the joints appears. According to Melamed et al. (1992), Type 1 and Type 2 DCS seem to be equally common. All in all, we have reasons to believe that the number of participants reporting DCI in our study is not an overestimation of the true number.

Some caution must also be made to the conclusion of adverse effects of DCI on neuropsychological functions as the several separate tests increase the probability of false positive findings. However, a correction for multiple testing using Bonferroni, or a less conservative approach such as the *q* value (Storey and Tibshirani 2003), still gives a significant result for an adverse effect of DCI on the Benton VRT Errors test. We chose not to present *p* values adjusted for multiple comparisons as this largely inflates the false negative error (Rothman 1990).

Another potential weakness could be that the neuropsychologist conducting the test was not blinded as regards the divers’ lifestyle parameters and health complaints including incidents of DCI. However, a neuropsychological examination is a very structured test, and the neuropsychologist (RB-P) was very experienced and conducted the tests in a standardized way. Other factors, like head injury, if unevenly distributed between the groups with and without incidents of DCI could also lead to a distortion in the observed association between the test results and DCI incidents.

The study was designed without an external control group. However, the large exposure contrasts within the group with respect to the number of dives allowed us to study the differences in test results related to the number of dives. In this respect, individuals with few dives serve the role of a control group better than an external control group which could introduce a possible difference in other sociodemographic characteristics.

## Conclusions

In this prospective study of young men, examined while they were students in a diving school for professional divers, most neuropsychological test results were remarkably stable over a 12-year period. No adverse effects were associated with the individuals’ cumulative number of dives. The divers with an incident of DCI reported more neuropsychiatric symptoms, and they performed worse in a memory test. None of the divers in the present study were involved in a litigation process, and there was no indication of a suboptimal performance motivated by external incentives among the divers with an incident of DCI.

The diver students who were able to remember most wooden shapes presented to them while blindfolded had an increased likelihood of becoming a professional diver 12 years later.

The observed associations between a history of DCI and impaired results in a memory test may be due to a nervous system effect caused by DCI. However, other hypothetically unmeasured predictors of this outcome cannot be ruled out.

The main findings in the present study support the view that asymptomatic non-saturation divers who have dived under controlled conditions do not have an increased risk of impaired nervous system function, at least not to an extent that can be detected with neuropsychological tests while they still are relatively young.
